# The Effect of Different Diluents and Curing Agents on the Performance of Epoxy Resin-Based Intumescent Flame-Retardant Coatings

**DOI:** 10.3390/ma17020348

**Published:** 2024-01-10

**Authors:** Xukun Yang, Yange Wan, Nan Yang, Yilin Hou, Dantong Chen, Jiachen Liu, Guoshuai Cai, Mingchao Wang

**Affiliations:** 1 School of Materials and Engineering, Key Lab of Advanced Ceramics and Machining Technology of Ministry of Education, Tianjin University, 92 Weijin Road, Tianjin 300072, China; xukun_yang@163.com (X.Y.); jcliu@tju.edu.cn (J.L.); 2 Beijing Institute of Astronautical Systems Engineering, Beijing 100076, China; 3 Department of Safety Engineering, Civil Aviation University of China, 2898 Jinbei Road, Tianjin 300300, China; wanyange2022@163.com (Y.W.); yyangnan11@163.com (N.Y.); ylhou1121@163.com (Y.H.); 4 College of Science, Civil Aviation University of China, 2898 Jinbei Road, Tianjin 300300, China; chendantong1110@163.com

**Keywords:** epoxy resin, flame-retardant insulation coating, diluent, curing agent, flame-retardant performance, mechanical properties

## Abstract

The epoxy resin-based (ESB) intumescent flame-retardant coatings were modified with 1,4-butanediol diglycidyl ether (14BDDE) and butyl glycidyl ether (BGE) as diluents and T403 and 4,4′-diaminodiphenylmethane (DDM) as curing agents, respectively. The effects of different diluents and curing agents on the flame-retardant and mechanical properties, as well as the composition evolution of the coatings, were investigated by using large-plate combustion, the limiting oxygen index (LOI), vertical combustion, a cone calorimeter, X-ray diffraction, FTIR analysis, a N_2_ adsorption and desorption test, a scanning electron microscope (SEM), a tensile strength test, and a viscosity test. The results showed that the addition of 14BBDE and T403 promoted the oxidation of B_4_C and the formation of boron-containing glass or ceramics, increased the residual mass of char, densified the surface char layer, and increased the specific surface area of porous residual char. When their dosage was 30%, ESB-1T-3 coating exhibited the most excellent flame-retardant properties. During the 2 h large-plate combustion test, the backside temperature was only 138.72 °C, without any melting pits. In addition, the peak heat release rate (PHRR), total heat release rate (THR), total smoke production (TSP), and peak smoke production (PSPR) were reduced by 13.15%, 13.9%, 5.48%, and 17.45%, respectively, compared to the blank ESB coating. The LOI value reached 33.4%, and the vertical combustion grade was V-0. In addition, the tensile strength of the ESB-1T-3 sample was increased by 10.94% compared to ESB. In contrast, the addition of BGE and DDM promoted the combustion of the coating, affected the ceramic process of the coating, seriously affected the formation of borosilicate glass, and exhibited poor flame retardancy. The backside temperature reached 190.93 °C after 2 h combustion. A unified rule is that as the amount of diluent and curing agent increases, the flame retardancy improves while the mechanical properties decrease. This work provides data support for the preparation and process optimization of resin-based coatings.

## 1. Introduction

Fire is a devastating natural disaster that can be very harmful. Fire not only destroys buildings but also threatens human lives. According to statistics, about 170,000 people lose their lives to fire every year [1]. Among them, urban fires are very important because of the large population in the city, and there are many tall buildings. Steel as the main material in the city-building, once a fire occurs, will quickly lose its mechanical properties [2], leading to the collapse of the building. Therefore, there is an urgent need for a means of fire protection to provide the necessary thermal protection. The traditional means of protection is to inject cooling circulating water into steel, but this method is very costly and complicated to operate. In contrast, applying flame-retardant coatings is an economical and effective method, which can effectively slow down the spread of fire, thereby making the building better withstand the attack of the fire.

According to the flame-retardant mechanism, the coatings can be divided into intumescent flame-retardant coatings and non-intumescent flame-retardant coatings. Non-intumescent flame-retardant coatings rely on their non-combustibility and large thickness to provide a good flame-retardant insulation effect. However, its larger thickness will generate a larger mass load. Furthermore, its surface integrity is poor, making it easy for moisture to penetrate the coating, thus the substrate is subjected to a certain degree of corrosion [3]. Intumescent flame-retardant coatings have good fire performance and are usually low in thickness, which acts as a thermal barrier by reducing the heat transfer of fire to the structure by generating an expanded char layer structure when exposed to heat [4].

For intumescent coatings, the different matrix materials used have very different properties [5,6,7,8]. Among them, epoxy resin as a kind of polymer thermosetting resin material has good film-forming, corrosion resistance, and electric insulation properties [9,10,11,12]. It also has many polar groups and highly active epoxy groups, and it can also maintain good adhesion with the surface of many materials at room temperature, such as steel, aluminum, and titanium alloy materials [13,14]. Therefore, epoxy resin is often used as the base material of intumescent flame-retardant coatings [15,16,17]. However, the poor heat resistance and high viscosity of epoxy resins prevent them from being used directly, and there is an urgent need to modify them. In this field, there has been sufficient research on the fire resistance of modified epoxy resin-based flame-retardant coatings. Chen et al. [18] used dimethyldioxysiloxane condensed with 3-glycosyloxypropyltrimethoxysiloxane (KH-560) to prepare a silicone-modified epoxy resin-based coating (SiR) and cured the resin with a nitrogen- and a phosphorus-containing flame retardant (PTDP), which was able to achieve a V-1 rating in UL-94 testing, and the LOI value could reach 31%. Riyazuddin et al. [19] prepared a novel melamine polymer (ETPMP) using nucleophilic substitution reaction and prepared epoxy resin-based flame-retardant coatings together with APP and CuO. The flame-retardant properties of the coatings changed significantly as soon as trace amounts of CuO were doped, with a 13% increase in LOI value and a UL-94 test rating of V-0. However, research has seldom been conducted on the effect of diluents and curing agents on epoxy resin-based coatings. The viscosity of a flame-retardant coating is a very important property. It determines whether it can be effectively applied to the surface of the substrate. If the viscosity of the coating is too high, it will not be applied easily, leading to greater economic losses, so diluting it is urgently needed.

Epoxy resins can be diluted by using different types of chemicals, including diluents, catalysts, and solvents. Among them, adding diluents is one of the most commonly used dilution methods. An epoxy diluent is a chemical used to control the viscosity and cure rate of an epoxy resin. It is usually a low molecular weight organic compound that disperses in the epoxy resin and reduces its viscosity, making it easier to apply. The common epoxy resin diluents include alcohols, ketones, esters, and ethers. The last type is a reactive diluent, which is a type of chemical that reacts with epoxy resins and accelerates their curing rate [20,21,22,23]. They are usually low molecular weight organic compounds containing reactive functional groups that react with the epoxy groups in the epoxy resin to become part of a cross-linked mesh system, thereby reducing cure shrinkage.

In our previous studies, we prepared a kind of silicon and boron-modified epoxy resin-based flame-retardant coating (ESB) by using silicon resin and boron carbide as modifiers, ammonium polyphosphate (APP) as dehydration catalysts, pentaerythritol (PER) as a charring agent, melamine (MEL) as a foaming agent, and sepiolite and palygorskite as fillers. Although the modified coatings have excellent flame-retardant and mechanical properties, there are some disadvantages associated with using a water bath heating method to dilute the epoxy resin matrix. Firstly, there is a potential loss of volatile components during the heating process, which can impact the composition and performance of the epoxy resin. Secondly, it may be relatively difficult to precisely control the temperature and time during the heating process, leading to uneven heating or overheating. Furthermore, the viscosity is still high and the curing time is too short. These reasons make the coating hard to apply in engineering operations. To improve the applicability of the epoxy resin-based coatings without damaging their flame retardancy, mechanical properties, and flame-retardancy mechanism, it is necessary to adjust the preparation process. As the main matrix material in ESB coatings, the viscosity of epoxy resin plays a crucial role in the viscosity of the entire coating. In this work, two different reactive diluents, 1,4-butanediyl diglycidyl ether (14BDDE) and butyl glycidyl ether (BGE), were applied to dilute the coating. Additionally, T403 and 4,4′-diaminodiphenylmethane (DDM) as two corresponding curing agents were used to cure the epoxy resin. The flame retardancy tests, mechanical property tests, and relevant characterization tests were conducted to detect the influence of adding different dilute and curing agents on the performance of epoxy resin-based coatings.

## 2. Materials and Methods

### 2.1. Materials

All raw materials were used as received without further process. Epoxy resin (EP-51) and 593 curing agent were purchased from Wanqian Chemical Co., Ltd., Jiangjin, China, Ammonium polyphosphate (APP, analytically pure (AR), degree of polymerization *n* > 1000), melamine (MEL, AR), and sepiolite (Si_12_Mg_8_O_30_(OH)_4_(OH_2_)_48_H_2_O, AR) were purchased from Aladdin Industrial Co., Ltd., Shanghai, China. Pentaerythritol (PER, AR) was purchased from Shangshan Chemical Co., Ltd., Jinan, China. Palygorskite (Si_8_O_20_Mg_5_(Al)(OH)_2_(H_2_O)_4_·4H_2_O) was purchased from Taihang Co., Ltd., Tianjin, China. A solvent-free MK silicone resin (SILRES^®^MK, power) with a (CH_3_-SiO_3/2_)_x_ basic structure was purchased from Wacker Chemie AG, Munich, Germany. Isopropanol (purity ≥99.7%, liquid) was purchased from Fuchen Chemical Reagent Co., Ltd., Tianjin, China. Boron carbide (B_4_C) was purchased from Zhengxing Abrasives Co., Ltd., Jilin, China. Butyl glycidyl ether (BGE, AR), 4,4′-diaminodiphenylmethane (DDM, AR), and 1,4-butanediol diglycidyl ether (14BDDE, AR) were purchased from Aladdin Industrial Co., Ltd., Shanghai, China. Polyetheramine T403 curing agent was purchased from Yingtai Composite Material Co., Ltd., Dongguan, China.

### 2.2. Preparation Process of ESB Coating

Without adding diluents, the blank epoxy resin-based coating was named ESB, and its specific formula is shown in Table 1. The process began by taking epoxy resin in a beaker and heating it in a water bath at 60 °C to obtain diluted solution 1. Then, a measured amount of APP, PER, and MEL was taken into a mortar and ground into a uniform powder. Next, sepiolite and palygorskite were added to the APP–PER–MEL mixture and combined with solution 1. The mixed slurry was further heated in a water bath at 60 °C and stirred for 15 min to obtain solution 2.

Meanwhile, a specific amount of SILRES^®^ MK resin and isopropyl alcohol (mass ratio = 3:5) was stirred at room temperature (RT) for 3 h to obtain a silicone resin named solution 3. Then, a given amount of solution 3 was added to solution 2, and the mixed liquid combination was stirred in a 60 °C water bath for 5–10 min to obtain solution 4. After that, an additional dosage of B_4_C was added to solution 4, and the resulting mixture was stirred for 10 min in a 60 °C water bath to obtain solution 5. Finally, a measured amount of 593 curing agent was added to solution 5, and the desired ESB coating was achieved after stirring for 2 min. It should be noted that it is necessary to apply ESB coating quickly to prevent premature curing.

### 2.3. Preparation Process of the Modified Coatings with 1,4-Butanediol Diglycidyl Ether

Firstly, a quantitative mass ratio of epoxy resin and 14BDDE was taken into a beaker based on Table 1, which was named solution 1 after stirring it vigorously for 15 min at RT. Likewise, the pre-ground uniform APP–PER–MEL powder was added to solution 1 together with sepiolite and palygorskite, which was stirred for 15 min at RT to obtain solution 2. After that, solution 3 was prepared in the same manner as mentioned above and added to solution 2 to obtain solution 4 after stirring for 5 min at RT. Similarly, a quantitative amount of B_4_C was added to solution 4 and stirred at RT for 10 min to obtain solution 5. Finally, the T403 curing agent was added to solution 5 and stirred for 2 min at RT to obtain the modified coatings. For the convenience of comparison, these samples were named ESB-1T-X, where X represents the contents, as shown in Table 1.

### 2.4. Preparation Process of the Modified Coatings with Butyl Glycidyl Ether

Epoxy resin was added into a beaker and heated to 35 °C in a water bath. Then, a quantitative amount of BGE was added and stirred for 15 min. After cooling at RT, solution 1 was obtained. Thereafter, the above processes were repeated to obtain solution 5. A quantitative amount of DDM was heated to 120 °C and was kept warm for 20 min, and then it was added to solution 5 at RT to obtain the modified coatings. These samples were named ESB-BD-X, where X represents the contents, as shown in Table 2.

### 2.5. Flame-Retardant Test of Coatings

The flame-retardant properties of the coatings were examined with a large-plate combustion test, LOI test, vertical combustion test, and cone calorimetry test.

The steel plate with a size of 100 mm × 100 mm × 1 mm was sanded and then wiped with an alcohol solution. The prepared coating was uniformly applied to the steel plate using a blade-coating machine, and the thickness of the coating was controlled at 1.5 mm. After curing at RT, the coated steel plate was used for the large-plate combustion test. The large-plate combustion test was carried out according to standard GB/T12441-2005 [24] A butane flame gun was used as the heat source. The length of the flame was controlled at 80 mm, and the temperature of the flame was maintained at 1300 °C. The test was conducted for 2 h. Five type-k thermocouples were mounted on the backside of the coated plate to capture temperature changes during the test; test results are averaged from data obtained from five type-k thermocouples. The LOI values of the samples (size: 130.0 mm × 6.5 mm × 3.2 mm, prepared by a pouring–forming method) were tested according to ASTM D2863-97 [24] standard test method by using a JF-6 oxygen index analyzer (Sanfeng, Changzhou, China); each sample was tested 5 times, and the results were taken as the average of the 5 tests. The vertical flame test was conducted with the HS-RS-5 apparatus (Hesheng, Shanghai, China), and the combustion level was graded according to the evaluation criteria (ASTM D3801 [24]). In the vertical flame test, each sample was tested five times, and the figures used in this paper were taken from the most representative photographs of each sample tested. The size of the test sample was 100 mm × 13 mm × 3 mm. The cone calorimetry test was carried out by using the Vouch 6180 (Vouch, Suzhou, China) instrument, with a radiant flux of 35 KW/m^2^ based on ISO 5660 standards [24]. The specification of the sample was 100 mm × 100 mm × 4 mm. In cone calorimetry tests, only one test was performed per sample.

### 2.6. Characterization of Coatings

The residual char samples obtained from the large pate combustion test and the coating samples, which were calcined at different temperatures, were tested by the X-ray diffraction (XRD) technique (Rigaku, Tokyo, Japan). The scanning range was 5°~75°, the wavelength of the X-ray was 0.154056 nm, and the scanning speed was 5°/min. A Thermo Scientific Nicolet iS20 FTIR spectrometer (Nicolet, Waltham, MA, USA) was utilized in conjunction with the XRD test to better understand the composition of coatings under different conditions, which covered a range of 400 cm^−1^~4000 cm^−1^. The microstructures on the outer surface and inside of the residual char structure after a large-plate combustion test were observed by using an S-4800 scanning electron microscope (Hitachi, Tokyo, Japan). N_2_ adsorption and desorption tests were carried out using ASAP 2460 (Micromeritics Co., Ltd., Norcross, GA, USA). The test samples were residual char obtained from cone calorimetry tests, which were firstly degassed at 150 °C for 3 h under a helium atmosphere. After that, they were analyzed by the Brunauer–Emmet–Teller (BET) and Barrett–Joyner–Halenda (BJH) models to determine the specific surface area and pore volume. TG-DSC tests were performed on the samples using an SDT Q600 (TA, New Castle, DE, USA) simultaneous thermal analyzer at a heating rate of 20 °C/min under an air atmosphere, and the mass of the tested samples was about 3 mg. Characterization tests for all samples were performed only once.

### 2.7. Viscosity and Tensile Properties Testing

After the preparation of each coating sample, the viscosity was measured by NDJ-1B rotational viscometer (Changji Geological Instrument Co., Ltd., Shanghai, China). The samples for the tensile test were prepared according to GB/T2567-2008 [8] using the pouring–forming method. The specification of the tensile test sample was dumbbell-shaped, with a total length of 200 mm, thickness of 4 mm, width of 2 mm at both ends, and width of 10 mm in the middle portion. The viscosity and tensile properties of each sample were tested 5 times, and the results were averaged for each test. The mechanical properties were measured by using the CMT4504 microcomputer-controlled electronic universal testing machine (MTS, Jinan, China). The test data were obtained from the average of five samples. The tensile strength (σ) was calculated from Equation (1):

(1) $$\sigma =\frac{p}{b\cdot h}$$

wherein p is the maximum load applied to the specimen, b is the width of the specimen at the break, and h is the thickness of the specimen at the break.

## 3. Results and Discussion

### 3.1. Flame-Retardant Properties

**Figure 1:**
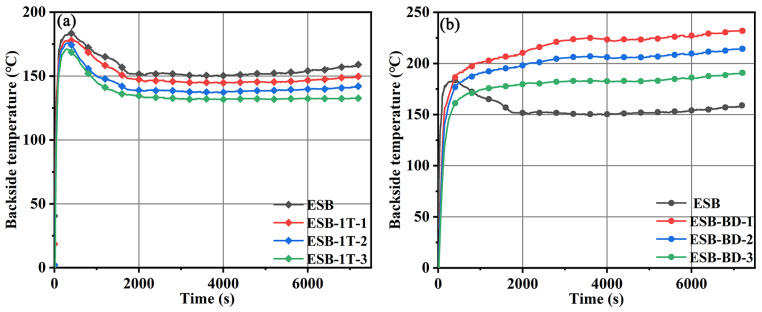
Temperature–time curves of different coatings during the large-plate combustion: (**a**) ESB-1T-X; (**b**) ESB-BD-X.

Figure 1 shows the backside temperature of different coatings during the large-plate combustion testing, and the key data are recorded in Table 3. The curves of the ESB coating can be divided into three stages. In the first stage, the temperature rises rapidly, which is mainly due to the direct contact of the coating with the hot flame. In the second stage, the temperature starts to decrease gradually, which is due to the expansion of the char layer structure by accessing heat. During this period, a large number of heat-absorbing reactions took place, which also played an important role in decreasing temperature. In the third stage, the backside temperature gradually stabilizes, which is due to the foam-like structure of the expanded char layer effectively insulating the external heat and controlling the temperature at a lower level.

As seen in Figure 1a, the temperature trends of ESB-1T-X coatings diluted with 14BDDE are all in a regular trend-like ESB. With the increase of 14BDDE doping, the overall backside temperature of ESB-1T-X coatings gradually decreases. Among them, the temperature of ESB-1T-3 is the lowest, with a maximum temperature of 170.27 °C and a final temperature of 138.72 °C, which were 7.34% and 15.59%, respectively, lower than the maximum (183.94 °C) and final (164.34 °C) temperatures of ESB. As seen in Figure 1b, the temperature trend of ESB-BD-X coatings modified with BGE dilution has changed, and its temperature rises rapidly at the beginning of the test, reaches the maximum value, and then gradually tends to stabilize. Throughout the entire process, there is no decrease in temperature. In addition, the temperature rises slightly at the end of the test, which is attributed to the cracks or voids on the surface of the char layer structure. On the other hand, as the doping amount of BGE increases, the temperature gradually decreases, but it is still higher than that of ESB coating. When 10 wt.% BGE dilution is added, ESB-BD-1 shows the worst flame-retardant performance. Although the maximum temperature (163.85 °C) of ESB-BD-3 coating in the first 500 s is 10.9% lower than that of ESB, its final temperature (190.93 °C) is 25.91% higher than that of ESB.

**Figure 2:**
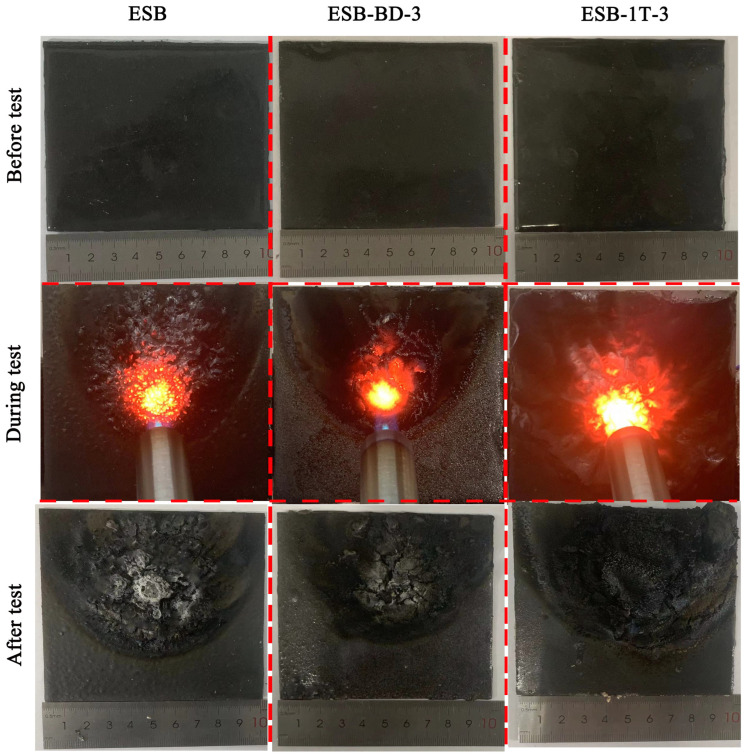
Physic photos of various coatings during the large-plate combustion test.

Figure 2 shows photographs of coatings resisting flame during the large-plate combustion test. Before combustion, all coatings maintain excellent integrity at the surface and no cracks appear. The surface of the char layer of ESB-1T-3 coating maintains high integrity during and after the combustion, while that of ESB-BD-3 coating shows obvious cracking. These cracks allow external heat sources to continuously enter the coating, leading to a gradual increase in temperature.

**Figure 3:**
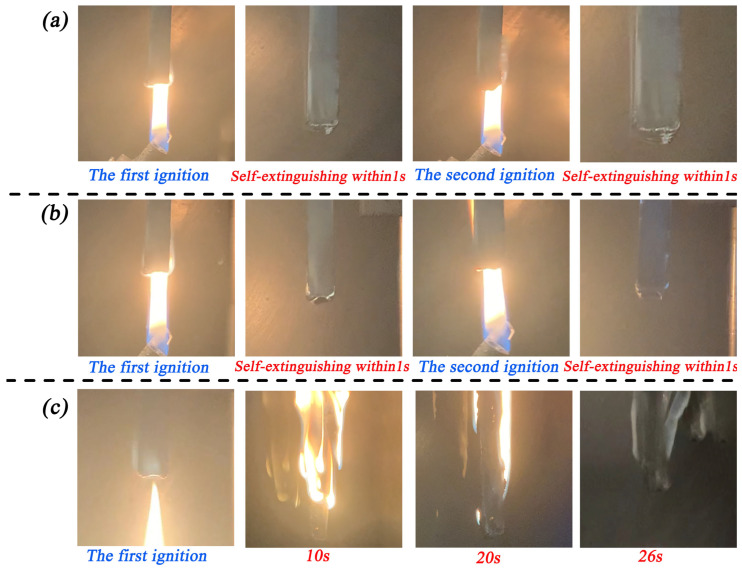
Physic photos of various coatings during the vertical combustion test: (**a**) ESB; (**b**) ESB-1T-3; (**c**) ESB-BD-3.

Figure 3 shows photographs of ESB, ESB-1T-3, and ESB-BD-3 coatings recorded during vertical combustion testing. It can be seen that ESB and ESB-1T-3 coatings are quickly extinguished within 1 s after two ignitions. Therefore, all of them belong to the V-0 level. ESB-BD-3 coating burns more violently after the initial ignition, and the burning lasts for a total of 24 s, which is rated as V-1 level according to the UL-94 evaluation ratings. Table 4 summarizes the data for vertical combustion and the LOI test. The LOI value of ESB-1T-3 coating was improved by 2.77% compared to ESB, while that of ESB-BD-3 was reduced by 11.07% compared to ESB.

### 3.2. Cone Calorimeter Test

**Figure 4:**
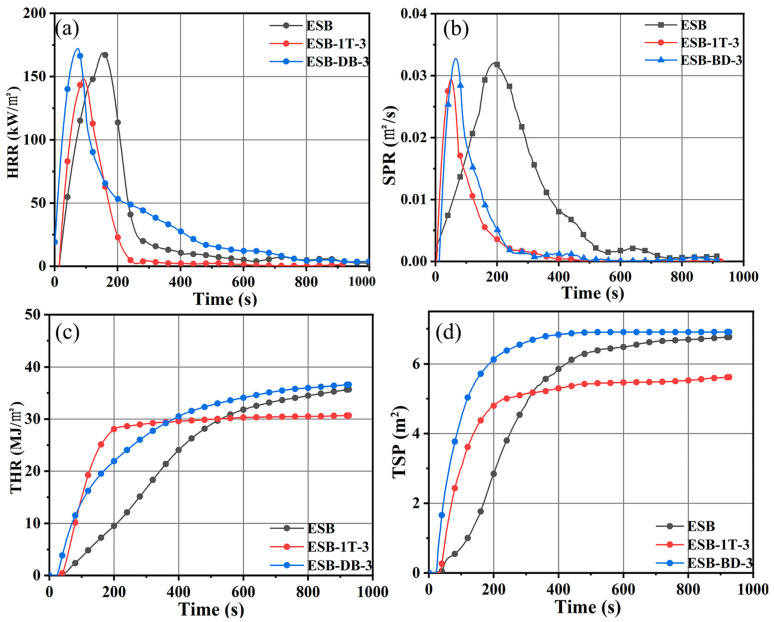
Cone calorimeter data for ESB, ESB-1T-3, and ESB-BD-3 coatings (**a**) HRR; (**b**) SPR; (**c**) THR; (**d**) TSP.

Figure 4 shows the heat release rate (HRR), smoke release rate (SPR), total heat release (THR), and total smoke release (TSP) curves of ESB, ESB-1T-3, and ESB-BD-3 coatings from the cone calorimeter test, and some specific data are listed in Table 5. Firstly, the peak heat release rate (PHRR) and THR values of ESB-1T-3 were the lowest at 148.1 kW/m^2^ and 30.67 MJ/m^2^, which were 13.15% and 13.9% lower than those of ESB, while the PHRR and THR values of ESB-BD-3 were 172.01 kW/m^2^ and 36.75 MJ/m^2^, which were 0.87% and 3.17% higher than those of ESB. A lower heat release indicates that the flame-retardant performance of the coating is better. Secondly, smoke emission is the second evaluation indicator. The peak smoke release rate (PSPR) and TSP values of ESB-1T-3 are also the lowest, which are 0.0293 m^2^/s and 5.62 m^2^, 5.48%, and 17.45%, respectively, lower than ESB. In contrast, the PSPR and TSP of ESB-BD-3 are 3.20% and 1.76%, respectively, higher than ESB.

**Figure 5:**
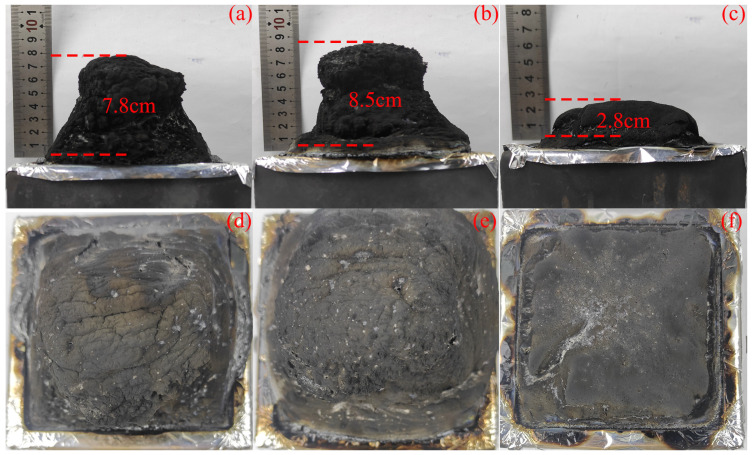
Digital physic photographs of the coatings after the cone calorimeter test (ESB: (**a**,**d**); ESB-1T-3: (**b**,**e**); ESB-BD-3: (**c**,**f**)).

Figure 5 shows the appearance photos of coatings after the conical calorimeter test. The residual char structure of ESB coating is in the form of a hill, which indicates that the coating has undergone significant volume expansion when exposed to heat. The structure of ESB-1T-3 coating is similar to ESB, but the height of the char layer is higher than that of ESB. On the contrary, the height of the expanded char layer of ESB-BD-3 coating is significantly lower than that of ESB. In addition, the residual mass of the ESB-1T-3 sample was 63.28%, which was 8.49% higher than that of ESB, while the mass residual of ESB-BD-3 was 39.47% lower than ESB. This phenomenon indicates that 14BDDE dilution and T403 curing agent increase the mass of residual char and enhance the expansion effect, while BGE dilution and DDM curing agent promote the erosion of the char layer and inhibit the formation of expanded char and hinder expansion.

From the above results, it can be seen that different diluents do indeed affect the flame-retardant and thermal insulation performance of the coating. The effect of combining 14BDDE dilution and T403 curing agent is much greater than that of combining BGE dilution and DDM curing agent. Also, the increase in the content of diluents and curing agents will improve the flame retardancy of the coating.

### 3.3. Composition Analysis of the Residual Char

**Figure 6:**
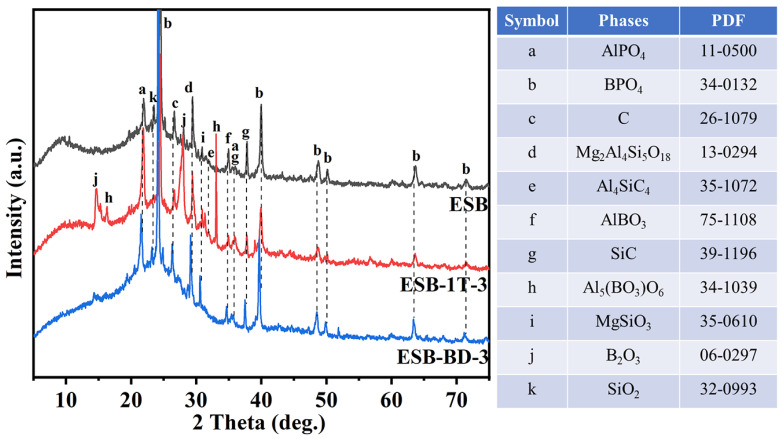
XRD spectra of the residual char of ESB, ESB-1T-3, and ESB-BD-3 coatings after combustion.

Figure 6 shows the XRD spectra of the residual char of ESB, ESB-1T-3, and ESB-BD-3 coatings after combustion. Two obvious amorphous broad peaks can be identified from the spectra of ESB. One broad peak appears at 2θ = 10°, which represents an amorphous SiO_2_ [25]. The amorphous SiO_2_ mainly comes from the decomposition of silicone resin, which can further improve the mechanical properties and ablation resistance of the residual char. The second broad peak appears between 20° and 40°, which is the amorphous char generated by the synergistic reaction between APP–PER–MEL and epoxy resin [26]. Some ceramic phases are identified on the spectrum, such as AlPO_4_, SiO_2_, MgSiO_3_, and Mg_2_Al_4_Si_5_O_18_, which come from the reaction of APP, palygorskite, and sepiolite at high temperatures. In addition, SiC and Al_4_SiC_4_ in the residual char are mainly formed from the char reduction of SiO_2_ and Al_2_O_3_; BPO_4_ and AlBO_3_ are derived from the reaction of B_2_O_3_ generated from the oxidation of B_4_C with HPO_3_, palygorskite, and sepiolite at elevated temperatures. The appearance of these ceramic phases also greatly improves the flame ablation resistance of the residual char.

Compared with ESB, the peaks of Al_5_(BO_3_)O_6_ and B_2_O_3_ are identified on the spectrum of ESB-1T-3. It is supposed that the addition of 14BDDE would promote the oxidation of B_4_C and the formation of aluminum borate. Also, the area of the broad peak at 10° has decreased slightly, which may be due to the reaction of some amorphous silica with boron to form glass. The in situ generation of boron-containing glass and ceramics can significantly improve the ablation resistance and thermal insulation effect of coatings. The XRD spectrum of the ESB-BD-3 coating does not change significantly compared with ESB, except for the disappearance of the broad peak at 10°, which implies that the addition of BGE dilution affected the ceramic transformation of silicone resin. With less residual char, the flame-retardant performance is very poor. The lower generation rate of SiO_2_ can be attributed to the introduction of BGE promoting the combustion of the resin, resulting in the volatilization of silicon at higher temperatures. In addition, a number of other ceramic phase substances are generated within the char layer. AlPO_4_ and MgSiO_3_ are formed through the crystallization reaction of palygorskite and sepiolite. Additionally, the process also results in the formation of crystalline SiO_2_ and Mg_2_Al_4_Si_5_O_18_. Moreover, the presence of char facilitates the reduction of silica and alumina, leading to the generation of SiC and Al_4_SiC_4_. AlBO_3_ and BPO_4_ were formed through the reaction between B_2_O_3_, HPO_3_, and Al_2_O_3_.

**Figure 7:**
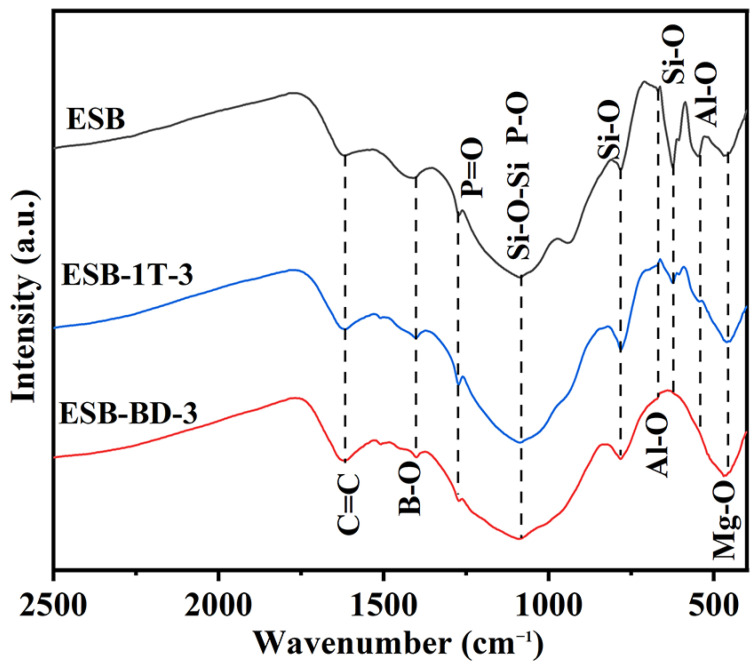
FTIR spectra of the residual char of ESB, ESB-1T-3, and ESB-BD-3 coatings after combustion.

Figure 7 shows the FTIR spectra of the residual char of the three coatings after combustion. The peaks at 1620 cm^−1^ appear in all spectra, which corresponds to the amorphous char that is the main component of the residual char. In addition, the appearance of the Si-O-Si peak at 1090 cm^−1^ indicates that the residual char contains amorphous silica. In addition, the absorption peaks of Si-O (455 cm^−1^, 798 cm^−1^), Mg-O (457 cm^−1^), Al-O (671 cm^−1^, 550 cm^−1^), and P=O (1270 cm^−1^) appear on all spectra, which corresponds to the presence of SiO_2_, MgSiO_3_, Mg_2_Al_4_Si_5_O_18_, and AlPO_4_ ceramics in the structural composition of the char layer. From the FTIR spectrum, it is hard to distinguish the difference in Si-O-Si bonds for the three coatings’ chars, but the B-O peak (1400 cm^−1^) almost disappears on the spectrum of ESB-BD-3, indicating a decrease in borosilicate content. The reason for this result is that a small amount of SiO_2_ cannot effectively immobilize boron oxide, resulting in excessive volatilization of boron oxide. Therefore, the addition of BGE not only affects the conversion of silica but also further causes the volatilization of boron oxide [27,28].

### 3.4. Composition Evolution of Coatings with Temperature

**Figure 8:**
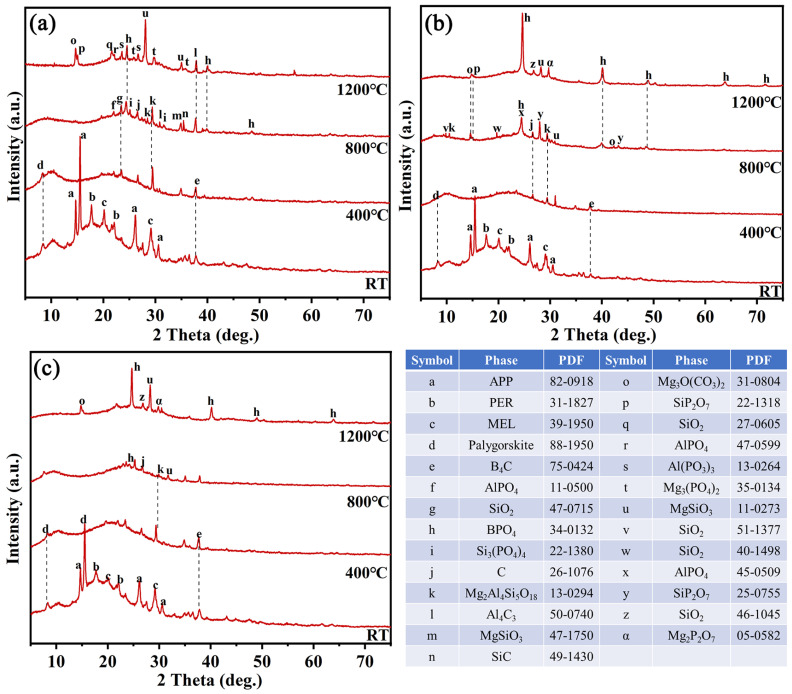
XRD spectra of ESB (**a**), ESB-1T-3 (**b**), and ESB-BD-3 (**c**) coatings after being calcined at different temperatures.

**Figure 9:**
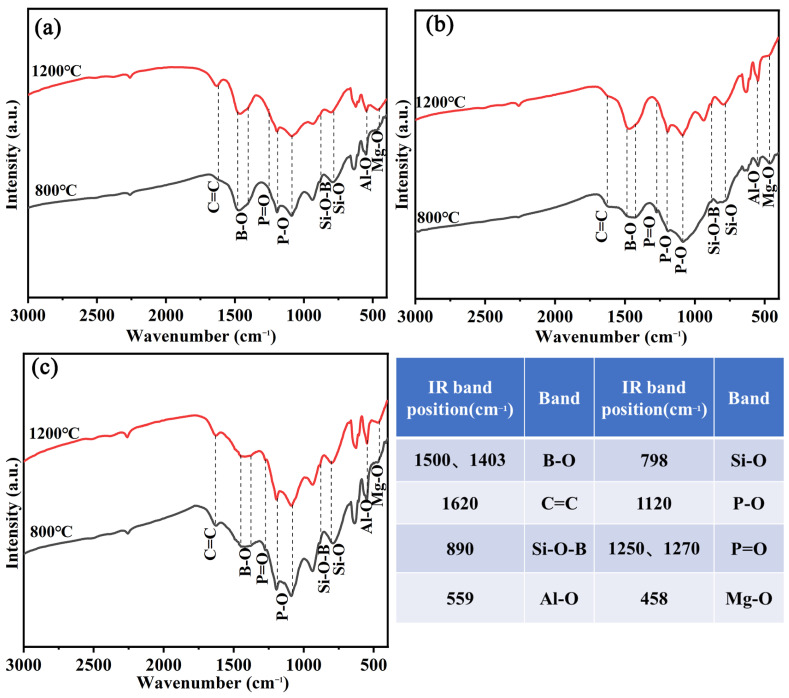
FTIR spectra of ESB (**a**), ESB-1T-3 (**b**), and ESB-BD-3 (**c**) coatings after being calcined at 800 °C and 1200 °C.

To better understand the addition of dilutions and curing agents on the composition evolution of coatings, the XRD spectra of ESB, ESB-1T-3, and ESB-BD-3 coatings after being calcined at 400 °C, 800 °C, and 1200 °C are tested. As seen from Figure 8a, in addition to the crystalline peaks of APP, PER, MEL, B_4_C, and palygorskite at room temperature, a clear amorphous broad peak between 15° and 35° corresponds to the epoxy resin [29], and another amorphous broad peak at 10° represents the silicone resin [30]. When the temperature rises to 400 °C, the amorphous broad peak of epoxy resin moves to the right, and the peaks of APP, MEL, and PER disappear, indicating that the first stage of reaction after encountering fire is almost complete at 400 °C. This stage mainly involves the rapid expansion of the coating and the internal endothermic reaction. The second stage within the range of 400 to 800 °C should belong to the early stage of ceramic transformation, during which the silicone resin begins to decompose into silica and boron carbide begins to be oxidized. After calcination at 800 °C, the broad peak at 10° moves a little to the left and the peak of B_4_C almost disappears. In addition, some intermediate ceramics begin to form at 800 °C. After treatment at 1200 °C, the coating exhibits extremely high ceramicization, and some heat-resistant ceramics are generated, such as SiP_2_O_7_, BPO_4_, AlPO_4_, MgSiO_3_, etc. In addition, a certain amount of borosilicate glass is also formed at 1200 °C, as the broad peak of amorphous SiO_2_ disappears on the XRD spectrum, and an iconic B-O absorption peak representing the presence of glass appears at 1200 °C, as seen in Figure 9a [31,32,33,34].

The composition evolution of ESB-1T-3 and ESB-BD-3 from RT to 800 °C is similar to that of ESB. The difference is that after adding diluents and initiators, the intermediate phase during the ceramic process decreases, and the composition of the final ceramic phase varies. BPO_4_, MgSiO_3_, and SiO_2_ are the main ceramic phases for them. In addition, according to the previous analysis, the introduction of BGE dilution and DDM curing agent will weaken the fixation of boron oxide, which is consistent with the experimental results in this section. As seen in Figure 9c, the absorption peak of B-O is significantly weaker than the others.

### 3.5. Thermal Properties of Coatings

**Figure 10:**
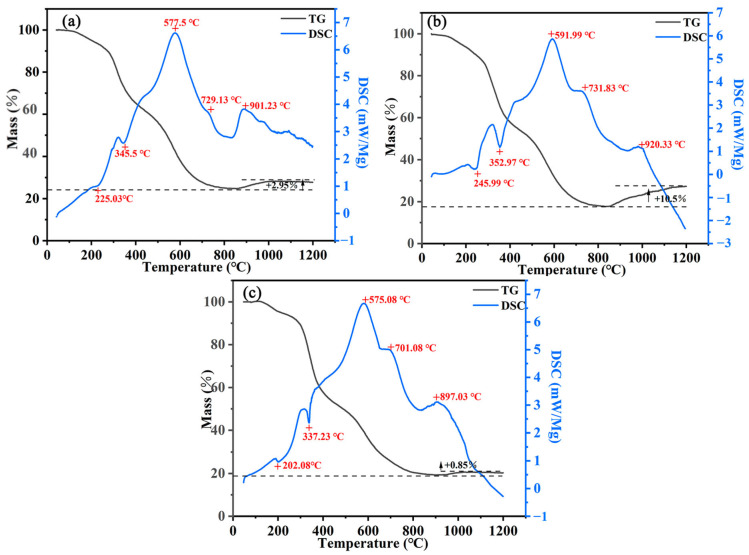
TG-DSC curves of various coatings: (**a**) ESB; (**b**) ESB-1T-3; (**c**) ESB-BD-3.

To further investigate the decomposition process and thermal stability of the coatings, TG-DSC tests are performed on ESB, ESB-1T-3, and ESB-BD-3 coatings, as shown in Figure 10. Table 6 records the key parameters of the experiment. As shown in Figure 10, the decomposition process of each sample can be divided into five stages, and the thermal weight loss in the first stage occurs at RT~250 °C. The thermal decomposition of the epoxy resin is the main cause of weight loss in this stage [35]. The corresponding heat absorption peak appears in the DSC curve, which is indicated by T_1_ in Table 6. It can be seen that the pyrolysis process of the epoxy resin in ESB-1T-3 coating is retarded compared with that of ESB. The epoxy groups and amino groups in 14BDDE and T403 can have a ring-opening reaction with the epoxy resin, which leads to the creation of a cross-linking bond with the epoxy resin and the formation of a three-dimensional network structure [36]. The cross-linking structure strengthens the physical and chemical properties of the epoxy resin, resulting in better heat resistance.

In contrast, the pyrolysis process occurs earlier in ESB-BD-3 coating than in ESB coating. Although BGE and DDM can also react with epoxy resin in a ring-opening reaction, the ESB-BD-3 coating is more flammable due to the lower flash point of BGE, which is about 54.4 °C (170.7 °C for 14BDDE). The second stage of pyrolysis occurs at 250 °C~450 °C, and the synergistic reaction between the APP–PER–MEL triad is the main reason for the weight loss in this process. The second endothermic peak in the DSC curve corresponds to this process, and the peak temperature is indicated by T_2_ in Table 6. The third stage of weight loss occurs from 450 °C to 600 °C. This stage of weight loss is caused by the ring-forming deconvolution of the siloxane units [37]. The heat exothermic peak in the DSC curve corresponds to this process, and the peak temperature is represented by T_3_ in Table 6. The weight loss in the fourth stage occurs between 600 °C and 800 °C, and the thermo-oxidative degradation of the residual char structure is the main cause of weight loss in this process. The exothermic peak in the DSC curve is relative to this process and is represented by T_4_ in Table 6. Unlike before, all samples experience varying degrees of increase in weight in the fifth stage, which is due to the oxidative expansion of B_4_C by heating. The exothermic peaks in the DSC curves are relative to this phase and are represented by T_5_ in Table 6. As seen in Figure 10, the ESB-1T-3 coating has a mass increase of 10.5% in this stage, which is about 255% higher than the mass increase of the ESB coating (2.95%), while the mass increase of the ESB-BD-3 coating (0.85%) is 71.19% lower than that of the ESB. It can be seen that the addition of 14BDDE with T403 contributes to the oxidative expansion process of B_4_C to produce more B_2_O_3_, which is finally converted into more borosilicate glass. In addition, B_2_O_3_ can effectively wet the internal pores of the residual char structure and optimize the shape of the pores, which improves the confinement of these closed pores. Therefore, the flame-retardant and heat insulation properties of ESB-1T-3 coating that were modified with 14BDDE and T403 are the best.

From Table 6, it can be seen that the temperatures of T_1_, T_2_, T_3_, T_4_, and T_5_ of the ESB-1T-3 coating are increased compared to ESB. Each pyrolysis process of the ESB-BD-3 coating occurs earlier. In addition, the mass remaining of ESB-1T-3 (27.33%) is increased by 2.16% compared with that of ESB (26.75%), while the mass remaining of ESB-BD-3 (20.29%) is decreased by 24.15% compared with that of ESB. This indicates that the thermal stability of the coating added with 14BDDE and T403 is improved.

### 3.6. Micro Morphology of the Residual Char Structures

**Figure 11:**
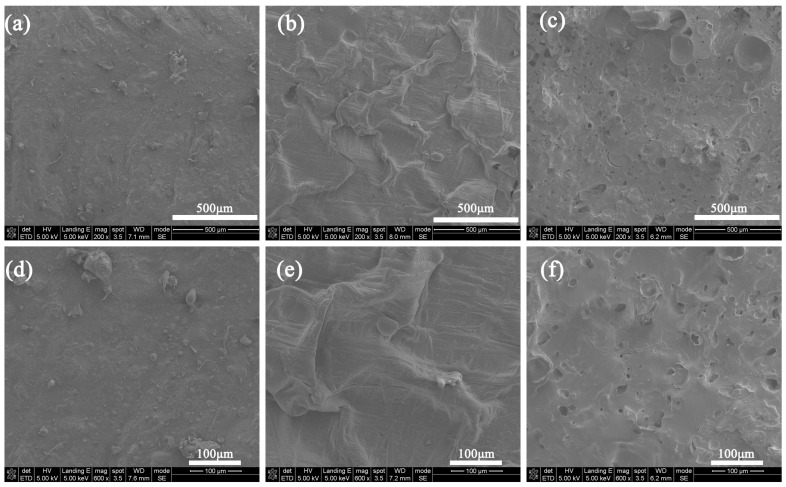
SEM images of the surface of the residual char structure after combustion: (**a**,**d**) ESB; (**b**,**e**) ESB-1T-3; (**c**,**f**) ESB-BD-3.

Figure 11 shows the SEM images of the resultant surfaces of three different residual chars. It can be seen in Figure 11a,d that the surface integrity of the ESB coating is high, with no cracks or holes appearing, which enables it to effectively resist the erosion of external heat sources and provide good thermal insulation. As seen in Figure 11b,e, the surface of the char layer for the ESB-1T-3 coating has a homogeneous fish-scale structure, which may be attributed to the melting of B_2_O_3_ as well as some borosilicate glass (see Figure 6). During the process of spraying fire, the surface layer of B_2_O_3_ can exert a certain cooling effect through volatilization, and the boron oxide seals the surface layer to prevent the invasion of flames, which also plays a certain role in improving the insulation effect of ESB-1T-3 coating. In contrast, a large number of holes have appeared on the surface of the char layer of the ESB-BD-3 coating (see Figure 11c,f). The appearance of these holes not only hinders the shielding of heat but also fails to effectively suppress the release of smoke inside the coating, resulting in a poor insulation effect and high smoke emission of the coating.

**Figure 12:**
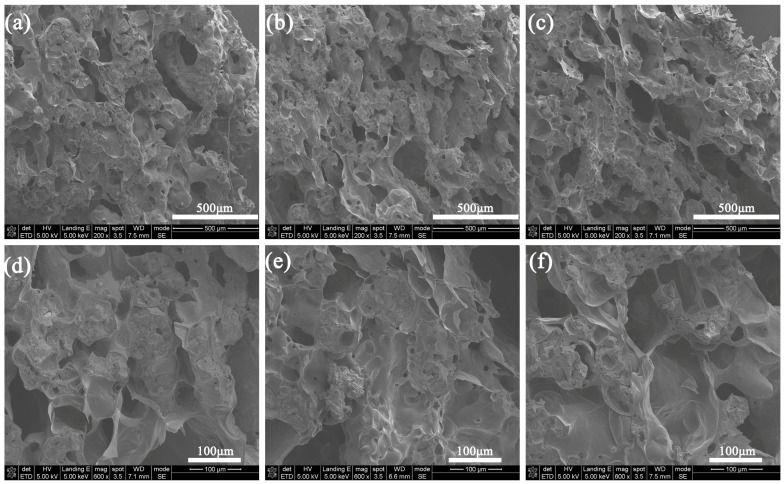
SEM images of the cross-section of the residual char structure after combustion: (**a**,**d**) ESB; (**b**,**e**) ESB-1T-3; (**c**,**f**) ESB-BD-3.

**Figure 13:**
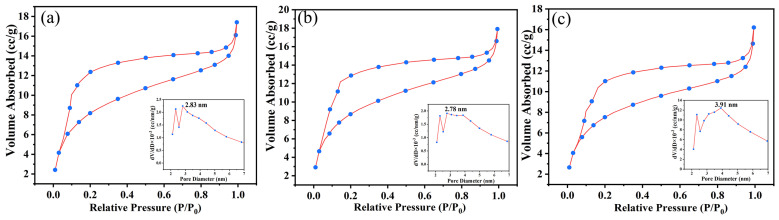
Isothermal adsorption and desorption curves for the residual char of different coatings: (**a**) ESB; (**b**) ESB-1T-3; (**c**) ESB-BD-3.

Figure 12 shows the internal SEM images of the residual char structure of the three coatings. The internal structure of the three types of residual char is not significantly different, and they are all porous structures, making it difficult to see their differences only from SEM images. To better understand these microstructures, a N_2_ adsorption and desorption test is applied to them. As shown in Figure 13, the adsorption and desorption curves of all three samples are of type IV (the IV curve indicates that the porous material has multiple adsorption forces on the surface, a pronounced pore structure, and a rapid increase in adsorption rate at low relative pressures), indicating the presence of mesoporous structures within the char layer structure [38,39,40]. Table 7 summarizes the detailed test data. The specific surface area value of the ESB-1T-3 is 32.23 m^2^/g, which is an improvement of 8.34% over the ESB. In addition, its pore size was reduced by 1.77% compared to ESB. For porous structures, a smaller pore size and a larger specific surface area are not only conducive to improving the thermal insulation ability of the char layer structure but also enhancing its mechanical strength [41]. In contrast, the specific surface area (28.57 m^2^/g) and pore size (3.91 nm) of the ESB-BD-3 sample have deteriorated to a certain extent compared with that of ESB, which is another reason for the poor insulation performance of ESB-BD-3.

### 3.7. Physical Performance

**Figure 14:**
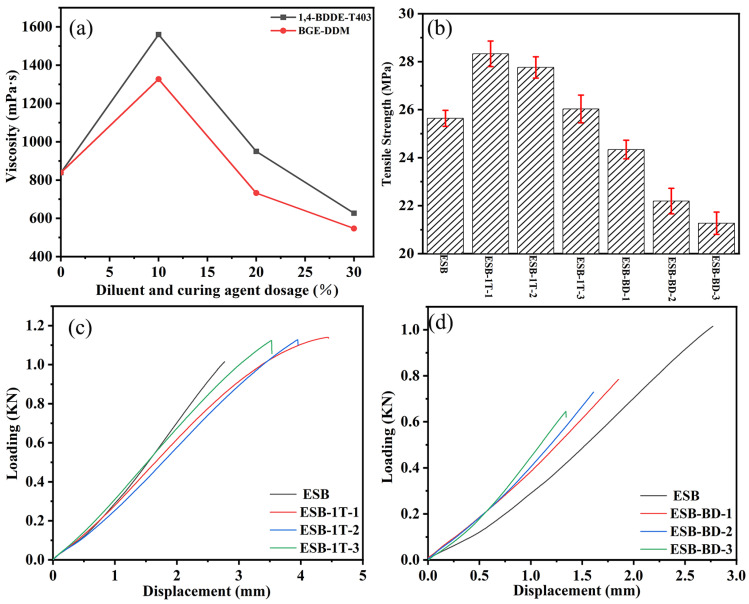
Physical and mechanical properties of various coatings: (**a**) viscosity; (**b**) tensile strength; (**c**,**d**) loading–displacement curves.

As shown in Figure 14a, the viscosity of the coating samples gradually decreased with the increase in the amount of dilute and curing agents. The viscosity of the two coatings, ESB-1T-3 and ESB-BD-3, reaches the lowest value, 627 mPa·s and 547 mPa·s, when the amount of dilute and curing agents doped are both 30%. The viscosity of the two coatings is 25.26% and 34.80% lower than that of ESB, and the lower viscosity is easy to brush or spray during the construction process.

Figure 14b shows the tensile properties of different coatings. The addition of 14BDDE and T403 increases the tensile strength, while the addition of BGE and DDM decreases the tensile strength. Regardless of the type of additive added, the tensile strength decreases with the increase of the amount added. Among them, ESB1T-1 has the highest tensile strength of 28.33 MPa, which is 10.49% higher than ESB; ESB-BD-3 has the lowest tensile strength of 21.27 MPa, which is 17.04% lower than ESB. Figure 14c,d show the loading–displacement curves for the tensile test. Similarly, the displacement values of all ESB-1T-X coatings are greater than that of ESB, indicating that the addition of 14BDDE and T403 can increase the toughness of coatings. In contrast, the addition of BGE and DDM makes the coating more brittle. The key physical and mechanical data are summarized in Table 7.

As seen from Table 8, the curing time of the ESB coating is only 0.6 h, which requires the coating to be quickly put into use after preparation, otherwise it results in economic losses. However, the curing time of ESB-1T-3 and ESB-BD-3 coatings has been effectively delayed, allowing for a certain period of storage after preparation and providing convenience for construction. The curing time of ESB-1T-3 and ESB-BD-3 coatings also falls within the requirements range specified in GB/T14907-2018 [5].

### 3.8. Comparison of the Comprehensive Performance of ESB-1T-3 and ESB-BD-3

Compared with ESB-BD-3, ESB-1T-3 has a better flame-retardant and heat insulation performance. A three-dimensional network structure formed from the ring-opening reaction between 14BDDE, T403, and epoxy resin can effectively improve the cross-linking density, which essentially improves the heat resistance and flame retardancy of the polymer matrix. According to the above analysis, the addition of 14BDDE and T403 also promotes the oxidative expansion of B_4_C, the formation of borosilicate glass, the increase of residual char mass, and the densification of a char layer structure, which is the key reason for ESB-1T-3 having a better flame-retardant and thermal insulation performance.

BGE and DDM can also react with epoxy resin in a ring-opening reaction. However, the flash point of BGE is too low, making the polymer matrix more prone to combustion. Moreover, the addition of BGE and DDM inhibits the oxidation of B_4_C in ESB-BD-3 coating, reduces the expansion effect, and makes the coating not able to generate enough borosilicate glass substances in the residual char structure after being exposed to fire. In addition, the residual mass of ESB-BD-3 is lower, and the pore structure has a low specific surface area. Therefore, ESB-BD-3 has weaker flame-retardant and heat insulation properties than ESB-1T-3.

The backside temperature of the substrate coated with ESB-1T-3 is 27.35% lower than that of the substrate coated with ESB-BD-3. The LOI value of ESB-1T-3 is 15.57% higher than that of ESB-BD-3, and the values of PHRR, THR, PSPR, and TSP for ESB-1T-3 are 16.14%, 19.82%, 10.92%, and 23.31% lower than those of ESB-BD-3. According to the standard UL-94, ESB-1T-3 is evaluated as V-0 grade, while ESB-BD-3 is V-2 grade. From the TG-DSC test, ESB-1T-3 also shows better thermal stability. When the temperature rises to 1200 °C, the mass residue of the ESB-1T-3 coating is 27.33%, which is 30.64% higher than ESB-BD-3.

Based on the mechanical property tests, the addition of 14BDDE and T403 improves the tensile strength of ESB coatings, while the addition of BGE and DDM reduces the tensile strength of ESB coatings. The mechanical properties of the coatings decrease with an increase in the amount of diluent and curing agents, which is contrary to the change ruler of the flame-retardant and thermal insulation performance. Therefore, the trade-off between flame retardancy and mechanical properties will have a profound effect on the practical and potential uses of epoxy-based intumescent flame-retardant coatings. If the flame-retardant property of an epoxy-based intumescent flame-retardant coating is too prominent, but the mechanical properties are too weak, the coating will be prone to deformation or rupture when subjected to stress, thus weakening the protective ability of the coating and affecting the flame-retardant effect of the coating in practical use. From the experimental data, ESB-1T-3 has excellent flame-retardant properties, and although the mechanical properties are not as good as ESB-1T-1 and ESB-1T-2, they are still better than the ESB coating, so ESB-1T-3 is chosen as the optimal sample in this study.

## 4. Conclusions

The epoxy resin-based (ESB) intumescent flame-retardant coatings were modified with 1,4-butanediol diglycidyl ether (14BDDE) and butyl glycidyl ether (BGE) as diluents and T403 and 4,4′-diaminodiphenylmethane (DDM) as curing agents, respectively. In the flame retardancy test, it was found that the coatings prepared with 14BDDE and T403 had good flame retardancy, among which ESB-1T-3 showed the best flame retardancy, and the backside temperature was only 138.72 °C after the 2 h large-plate combustion test. The peak heat release rate (PHRR), total heat release rate (THR), total smoke production (TSP), and peak smoke production (PSPR) were reduced by 13.15%, 13.9%, 17.45%, and 5.48%, respectively, compared to the blank ESB coating. Its LOI value was 33.4%, an improvement of 2.77% over ESB, with a vertical combustion rating of V-0. The residual char mass was improved by 8.49% compared to the ESB coating. The better flame retardancy brought by 14BBDE and T403 comes from the fact that their addition promoted the oxidation of B_4_C and the formation of boron-containing glass or ceramics, increased the residual mass of char, densified the surface char layer, and increased the specific surface area of porous residual char. Also, the tensile strength of ESB-1T-3 coating was 10.94% higher than ESB coating.

In contrast, the addition of BGE and DDM promoted the combustion of the coating, affected the ceramic process of the coating, seriously affected the formation of borosilicate glass, and exhibited poor flame retardancy. The backside temperature of ESB-BD-3 reached 190.93 °C after 2 h combustion, and the PHRR, THR, TSP, and PSPR were 0.87%, 3.17%, 1.76%, and 3.20% higher than ESB. The LOI value was 28.9%, and the vertical combustion grade was V-1 grade. After combustion, the residual mass of char of ESB-BD-3 was 39.47% lower than that of ESB. In addition, the tensile strength of ESB-BD-X coatings was lower than the blank ESB.

## Figures and Tables

**Table 1:** The specific formulas for different ESB-1T-X coatings (wt. %).

Samples	E-51	14BDDE	APP:PER:MEL (5:2:3)	Palygorskit:Sepiolite (1:1)	B_4_C	MK Resin	593	T403
ESB	65	0	16	5	3	10	20	0
ESB-1T-1	65	10	16	5	3	10	0	10
ESB-1T-2	65	20	16	5	3	10	0	20
ESB-1T-3	65	30	16	5	3	10	0	30

**Table 2:** The specific formulas for different ESB-BD-X coatings (wt. %).

Samples	E-51	BGE	APP:PER:MEL (5:2:3)	Palygorskit:Sepiolite (1:1)	B_4_C	MK Resin	593	DDM
ESB	65	0	16	5	3	10	20	0
ESB-BD-1	65	10	16	5	3	10	0	10
ESB-BD-2	65	20	16	5	3	10	0	20
ESB-BD-3	65	30	16	5	3	10	0	30

**Table 3:** The critical data of a large-plate combustion test for various coatings.

Samples	ESB	ESB-1T-1	ESB-1T-2	ESB-1T-3	ESB-BD-1	ESB-BD-2	ESB-BD-3
Peak temperature in the first 500 s (°C)	183.94	177.37	173.54	170.27	197.23	187.10	163.85
Final temperature (°C)	164.34	149.66	141.98	138.72	231.89	214.34	190.93

**Table 4:** UL-94 and LOI data for various coatings.

Samples	ESB	ESB-1T-3	ESB-BD-3
LOI (%)	32.5	33.4	28.9
UL-94 level	V-0	V-0	V-1

**Table 5:** Cone calorimeter data.

Samples	TTI (s)	PHRR (kW/m^2^)	THR (MJ/m^2^)	PSPR (m^2^/s)	TSP (m^2^)	Residual Mass (%)
ESB	29	170.53	35.62	0.0318	6.81	58.32
ESB-BD-3	30	172.01	36.75	0.0325	6.93	35.34
ESB-1T-3	22	148.10	30.67	0.0293	5.62	63.28

**Table 6:** Relevant parameters for TG-DSC tests.

Sample	T_1_ (°C)	T_2_ (°C)	T_3_ (°C)	T_4_ (°C)	T_5_ (°C)	Mass Residue (%)
ESB	225.03	345.5	577.5	729.13	901.23	26.75
ESB-1T-3	245.99	352.97	591.99	731.83	920.33	27.33
ESB-BD-3	202.08	337.23	573.08	701.08	897.03	20.29

**Table 7:** Parameters of the coatings after combustion.

Samples	ESB	ESB-1T-3	ESB-BD-3
BET surface area (m^2^/g)	29.75	32.23	28.57
Total pore volume of adsorption (cm^3^/g)	0.062	0.073	0.057
Pore size (nm)	2.83	2.78	3.91

**Table 8:** Physical property data for different coatings.

Sample	ESB	ESB-1T-1	ESB-1T-2	ESB-1T-3	ESB-BD-1	ESB-BD-2	ESB-BD-3
Viscosity (mPa·s)	839	1560	950	627	1327	732	547
Curing time (h)	0.6	35	20	10	40	27	18
Tensile strength (MPa)	25.64	28.33	27.76	26.03	24.34	22.19	21.27
Maximum Loading (KN)	1.015	1.139	1.128	1.123	0.783	0.729	0.645

## Data Availability

Data are contained within the article.
